# The association of cardiovascular risk factors with saturated fatty acids and fatty acid desaturase indices in erythrocyte in middle-aged Korean adults

**DOI:** 10.1186/s12944-015-0135-x

**Published:** 2015-10-24

**Authors:** Seung Rye Kim, So Yeon Jeon, Seung-Min Lee

**Affiliations:** Program of Clinical Nutrition, Graduate School of Human Environmental Sciences, Yonsei University, Seoul, South Korea; Department of Food and Nutrition, College of Human Ecology, Yonsei University, Seoul, South Korea

**Keywords:** Fatty acid composition, Erythrocyte, Cardiovascular risk, Delta 9 desaturase index, Delta 6 desaturase index

## Abstract

**Background:**

The quality of dietary fats is associated with risk of cardiovascular diseases (CVD). We aimed to investigate the association between fatty acids in erythrocyte membrane phospholipids and CVD risk factors in middle-aged Korean adults.

**Methods:**

Fifty-five middle-aged adults who underwent health examinations were included in this retrospective and cross-sectional study. Anthropometry, serum lipids, clinical parameters, and erythrocyte membrane phospholipid fatty acid data were obtained from a registry.

**Results:**

The proportion of C14:0 and C16:0 was greatly elevated in high quartile groups for triglyceride (TG) and systolic and diastolic blood pressure groups (SBP and DBP) (*p* = 0.042, *p* = 0.021, or *p* = 0.008 respectively) compared to low quartile groups. While C16:1n7 and/or C18:1n9 were positively associated with CVD risk factors, the delta 9 desaturase activity index (D9D) (C18:1n9/C18:0) was only significantly increased in high quartile groups for TG and blood pressures (*p* = 0.001, *p* = 0.002 or *p* = 0.003). Conversely, TG and blood pressures showed inverse relationships with C20:4n6 or D5D (C20:4n6/C20:3n6). C18:3n6 and/or D6D (C18:3n6/C18:2n6) were positively associated with insulin resistance and diabetic parameters. Particularly high D6D was detected in high quartile groups of FBS and insulin (*p* = 0.016 and *p* = 0.042). In linear regression analysis, D9D and/or C14:0 + C16:0 were significant contributors to serum TG and blood pressures. D6D was a contributing factor to FBS.

**Conclusions:**

The indices of D9D and D6D from erythrocyte membrane phospholipids and the proportion of saturated fatty acids were increased as the cardiovascular risk factors, including serum TG, blood pressures, and FBS increased their levels (IRB number C2014199 (1396)).

**Electronic supplementary material:**

The online version of this article (doi:10.1186/s12944-015-0135-x) contains supplementary material, which is available to authorized users.

## Introduction

Cardiovascular disorders (CVD) is especially prevalent in developing countries, including Korea, due to rapid dietary changes and Western style diets. It is well known that the quality of dietary fats, such as saturated fats, determines the risk of developing CVD [1]. For instance, dietary intake of saturated fatty acids (SFA) was closely associated with elevated blood cholesterols and increased risk of cardiovascular diseases [2]. Dietary polyunsaturated fatty acids (PUFAs) had an inverse relationship with the incidence of coronary heart diseases [2]. Thus, earlier detection of changes in dietary fat-associated CVD risk would be beneficial for preventing CVD development.

The type of fats in diets affects the composition of fatty acids in human bodies, including in blood [3]. Many studies have identified significant alterations at in the fatty acid patterns associated with CVD [4–9]. Changes in the composition of individual fatty acids and the levels of fatty acid desaturase are frequently assessed together to understand overall changes in fatty acid patterns [10]. Although many studies have tried to identify changes in fatty acid patterns in patients with CVD or associated disorders, there are only a limited number of studies investigating the association between fatty acids and/or fatty acid desaturase indices with individual risk components of CVD [11]. Knowledge of a specific association between fatty acid indices and individual risk factors would allow detection of alterations that could contribute to CVD.

In many cases, either plasma or serum was used to investigate the composition of fatty acids relationships with CVD [4–9]. Fatty acids in plasma or serum phospholipids reflect changes in dietary intake within a few days [12]. Erythrocyte membranes reflect long-term dietary changes that accumulate during the life time of erythrocytes [13]. In this study, we aimed to investigate any significant association between fatty acids from erythrocyte membrane phospholipids and CVD risk factors in middle-aged Korean men. This study was a retrospective cross-sectional study using data from patients who underwent a special health check-up consisting of anthropometrical measurements, blood chemistry, and analysis of erythrocyte membrane phospholipid fatty acid composition.

## Results

### Anthropometry, blood lipid profiles, and fatty acid composition of erythrocyte membrane phospholipids of study subjects

The general characteristics of study subjects (35 males and 22 females) are shown in Table 1. Cardiovascular risk factors used for the study included metabolic risk factors (waist circumference, TG, HDL-C, fasting blood glucose, systolic and diastolic blood pressures), diabetes-related factors (insulin, HbA1c, HOMA-IR), and heart disease-related factors (AI, hsCRP, homocysteine, ApoB). The average values of age, systolic blood pressure (SBP), diastolic blood pressure (DBP), total-C, LDL-C, apolipoprotein B, insulin, hs-CRP, and HbA1c were not different between men and women (Table 1). BMI, serum TG, FBS, insulin, and homocysteine levels were slightly higher in men than in women. HDL-C was higher in women than in men (Table 1). In terms of dietary intake, total alcohol and calorie intake were higher in men than in women (Table 1). There were no significant differences in the percentages of fatty acids in erythrocyte membrane phospholipids between men and women except for linoleic acid (LA, C18:2n6) and lignoceric acid (C24:0) (Additional file 1: Table S1). In women, LA was slightly higher and lignoceric acid was lower compared to men (*p* = 0.014 and *p* = 0.007, respectively) (Additional file 1: Table S1). A total of 24.6 % of study subjects had central obesity, 28.1 % had hypertriglyceridemia, 21.1 % had low HDL-C, 17.5 % had impaired fasting blood glucose, and 21.1 % had hypertension according to the criteria for metabolic syndrome risk factors.

**Table 1:** General characteristics of study subjects

	Total	Male	Female	Male–female
	(*n* = 57)	(*n* = 35)	(*n* = 22)	*p*-value
Age, years	52.56 ± 4.751	53.43 ± 4.231	51.18 ± 5.288	0.082
BMI, kg/m2	23.55 ± 2.624	24.47 ± 2.295	22.10 ± 2.496	0.001
SBP, mmHg	116.4 ± 16.93	120.0 ± 16.84	110.8 ± 15.87	0.046
DBP, mmHg	69.91 ± 11.68	72.17 ± 11.95	66.32 ± 10.50	0.065
TG, mg/dL	117.5 ± 61.40	131.0 ± 54.67	96.00 ± 66.51	0.035
TC, mg/dL	200.8 ± 33.97	195.3 ± 33.97	209.4 ± 32.90	0.129
HDL-C, mg/dL	53.51 ± 10.96	49.94 ± 8.691	59.18 ± 11.95	0.001
LDL-C, mg/dL	123.7 ± 30.55	119.3 ± 31.14	130.7 ± 28.90	0.171
AI	2.848 ± 0.768	2.966 ± 0.680	2.611 ± 0.857	0.142
Apo B, g/L	0.985 ± 0.186	0.998 ± 0.178	0.964 ± 0.201	0.512
FBS, mg/dl	100.7 ± 13.68	104.7 ± 14.98	94.32 ± 8.156	0.004
Insulin, μIU/ml	6.486 ± 3.833	7.178 ± 4.225	5.274 ± 2.718	0.076
HOMA-IR	1.591 ± 1.090	1.886 ± 1.183	1.122 ± 0.726	0.004
Homocysteine, μmol/L	10.44 ± 3.393	11.85 ± 3.343	8.195 ± 2.006	<0.001
HbA1c, %	5.472 ± 0.366	5.543 ± 0.414	5.359 ± 0.240	0.064
hs-CRP, mg/L	0.686 ± 0.597	0.708 ± 0.667	0.652 ± 0.477	0.736
Alcohol intake, wk	66.67 ± 61.94	98.04 ± 54.88	16.77 ± 33.24	<0.001
Total energy intake, %	98.63 ± 18.61	92.54 ± 14.10	108.3 ± 21.01	0.004
Carbohydrate intake, %	90.89 ± 34.92	88.51 ± 41.71	94.68 ± 20.25	0.521
Protein intake, %	109.2 ± 34.01	104.2 ± 32.28	117.0 ± 35.94	0.171
Fat intake, %	126.5 ± 45.45	113.0 ± 40.57	147.8 ± 45.44	0.004
Metabolic syndrome, n (%)	10 (17.54)	8 (22.86)	2 (9.091)	
Central obesity, n (%)	14 (24.56)	10 (28.57)	4 (18.18)	
Hypertriglyceridemia, n (%)	16 (28.07)	12 (34.29)	4 (18.18)	
Low HDL-C, n (%)	12 (21.05)	7 (20.00)	5 (22.73)	
Impaired fasting glucose, n (%)	10 (17.54)	9 (25.71)	1 (4.545)	
Hypertension, n (%)	12 (21.05)	9 (25.71)	3 (13.64)	
Postmenopausal, n (%)	10 (45.45)	–	10 (45.45)	

*Mean ± S.D.* tested by independent t test, *BMI* body mass index, *SBP* Systolic Blood Pressure, *DBP* Diastolic Blood Pressure, *TG* triglyceride, *TC* total cholesterol, *HDL-C* high density lipoprotein cholesterol, *LDL-C* low density lipoprotein, cholesterol, *AI* (TC-HDL)/HDL, *Apo B* apolipoprotein B, *FBS* fasting blood sugar, *HOMA-IR* homeostasis model assessment of insulin resistance, *hs-CRP* high sensitivity C-reactive protein; intake % percentage of recommended intake and the actual intake

### Correlation between erythrocyte membrane phospholipid fatty acids and cardiovascular risk factors

**Fig. 1:**
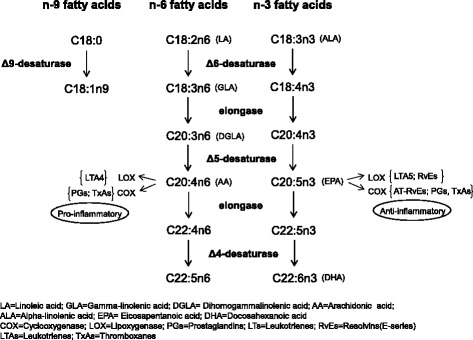
The pathway of the fatty acids and related fatty acid desaturases

The levels of cardiovascular risk factors were investigated for their correlations with the proportions of fatty acids in erythrocyte membrane phospholipids (Table 2). The pathway of the fatty acids was shown in Fig. 1. Among saturated fatty acids (SFA), myristic acid (C14:0) and palmitic acid (C16:0) were positively correlated with waist circumference, TG, SBP, DBP, FBS, insulin, HOMA-IR, and AI and were negatively correlated with HDL-C. Stearic acid (C18:0) showed a negative correlation with TG, SBP, and DBP. Of monounsaturated fatty acids (MUFA), palmitoleic acid (C16:1n7) and oleic acid (C18:1n9) were positively related to TG, SBP, DBP, insulin, HOMA-IR, ApoB, and homocysteine. Eicosenoic acid (C20:1n9) and nervonic acid (C24:1n9) were negatively related to TG, SBP, and DBP. Of n-6 polyunsaturated fatty acids (PUFA), γ–linolenic acid (GLA, C18:3n6) was positively correlated with TG, FBS, insulin, AI, and HOMA-IR while arachidonic acid (AA, C20:4n6) was negatively correlated with TG, SBP, DBP, and apoB. Docosapentaenoic acid (C22:5n6) was negatively related to HbA1c. The n-3 PUFAs did not show correlation with study factors. Desaturase activity indices including D9D, D6D, and D5D were examined for their correlation with proportions of fatty acids. D9D (C18:1n9 /C18:0) and D6D (C18:3n6/C18:2n6) were positively associated with TG, SBP, DBP, FBS, insulin, HbA1c, HOMA-IR, and apoB. D5D was negatively associated with TG, SBP, DBP, AI, and apoB. Overall, the findings indicated that cardiovascular risk factors were positively associated with the percentages of C14:0 and C16:0, C16:1n7 and C18:1n9 were associated with D9D, and C18:3n6 and D6D were negatively associated with C20:4n6 and D5D.

**Table 2:** Correlation analysis between erythrocyte membrane phospholipid fatty acids and cardiovascular risk factors

	BMI	Waist circumference	TG	Apo B	TC	LDL-C	HDL-C	SBP	DBP	FBS	Insulin	AI	HbA1c	HOMA-IR	homocysteine	hs-CRP
C14:0 Myristic acid	0.283^*^	0.285^*^	0.480^**^	0.130	−0.053	−0.100	−0.322^*^	0.150	0.177	0.308^*^	0.429^**^	0.343^**^	0.173	0.457^**^	0.145	0.088
C16:0 Palmitic acid	0.256	0.279^*^	0.375^**^	−0.007	−0.198	−0.299^*^	−0.297^*^	0.480^**^	0.496^**^	0.146	0.129	0.122	0.000	0.209	0.033	0.139
C18:0 Stearic acid	0.043	0.059	−0.341^**^	−0.189	−0.058	0.032	0.100	−0.326^*^	−0.329^*^	−0.150	−0.011	−0.205	−0.004	−0.064	−0.122	−0.038
C24:0 Lignoceric acid	−0.041	0.134	−0.058	−0.075	−0.015	0.061	0.044	−0.176	−0.221	0.163	−0.085	−0.053	0.043	−0.037	0.138	−0.311^*^
C16:1n7, Palmitoleic acid	0.059	−0.022	0.454^**^	0.114	0.091	−0.079	0.023	0.140	0.209	0.228	0.098	0.078	−0.008	0.123	0.088	0.122
C18:1n9, Oleic acid	0.099	0.213	0.523^**^	0.345^**^	0.021	−0.068	−0.157	.0387^**^	0.407^**^	0.033	0.269^*^	0.212	0.034	0.274^*^	0.269^*^	0.110
C20:1n9, Eicosenoic acid	0.093	0.043	−0.328^*^	−0.107	−0.156	0.022	−0.127	−0.288^*^	−0.249	0.072	0.054	0.012	0.171	0.076	−0.184	0.115
C24:1n9, Nervonic acid	−0.057	0.003	−0.227	−0.077	0.048	0.160	0.106	−0.316^*^	−0.338^*^	0.098	−0.056	−0.020	0.105	−0.039	0.115	−0.201
C18:2n6 (LA)	−0.214	−0.335^*^	−0.179	0.066	0.364^**^	0.376^**^	0.216	−0.052	−0.069	−0.245	−0.109	0.013	−0.123	−0.195	−0.145	0.037
C18:3n6 (GLA)	0.125	0.090	0.359^**^	0.085	0.049	0.033	−0.219	0.018	0.044	0.324^*^	0.349^**^	0.265^*^	0.250	0.286^*^	0.029	0.041
C20:2n6 (EDA)	−0.136	−0.140	−0.092	0.051	0.104	0.005	0.283^*^	−0.096	−0.087	−0.113	−0.056	−0.248	0.055	−0.038	0.079	−0.042
C20:3n6 (DGLA)	0.168	0.080	0.065	0.127	0.095	0.149	−0.160	0.212	0.241	0.046	0.163	0.181	0.209	0.137	0.127	0.245
C20:4n6 (AA)	−0.080	−0.020	−0.469^**^	−0.284^*^	−0.159	0.012	0.004	−0.284^*^	−0.276^*^	−0.034	−0.188	−0.201	0.108	−0.220	−0.051	0.005
C22:4n6 (DTA)	0.165	0.147	−0.244	−0.147	−0.084	0.065	−0.140	−0.027	−0.011	0.145	0.165	−0.037	0.131	0.130	0.003	0.064
C22:5n6 (DPA)	−0.178	−0.104	0.128	0.199	0.287^*^	0.245	0.166	−0.032	−0.090	−0.174	−0.165	−0.001	−0.265^*^	−0.224	0.048	−0.006
C18:3n3 (ALNA)	0.024	−0.057	0.215	0.011	−0.029	−0.081	−0.048	0.085	0.050	0.043	0.087	0.060	−0.168	−0.012	−0.162	0.123
C20:5n3 (EPA)	0.181	0.202	−0.166	−0.113	−0.221	−0.237	−0.041	0.017	0.032	0.138	−0.035	−0.053	0.063	0.055	0.063	−0.150
C22:5n3 (DPA)	0.182	0.229	−0.185	−0.224	−0.316^*^	−0.247	−0.151	−0.091	−0.090	0.185	−0.005	−0.096	0.048	0.072	−0.093	−0.108
C22:6n3 (DHA)	0.874	0.075	0.039	−0.117	−0.143	−0.227	−0.107	0.044	0.036	0.114	−0.147	0.008	0.011	−0.082	–.0810	–.0582
D5D	−0.233	−0.126	−0.035^**^	−0.291^*^	−0.179	−0.117	0.158	−0.366^**^	−0.400^**^	−0.056	−0.248	−0.302^*^	−0.155	−0.240	−0.179	−0.228
D6D	0.191	0.191	0.365^**^	0.048	−0.070	−0.078	−0.284^*^	0.003	0.049	0.383^**^	0.364^**^	0.089	0.303^*^	0.330^*^	0.087	0.044
D9D	0.042	0.119	0.546^**^	0.318^*^	0.016	−0.097	−0.167	0.453^**^	0.459^**^	0.060	0.179	0.226	−0.020	0.196	0.236	0.127

Mean ± S.D.; Significantly correlated by pearson correlation analysis at *p <0.05, and **p <0.01 and respectively

*AA* Arachidonic acid, *ALNA* α-Linolenic acid, *DGLA* Dihomo-γ-linolenic acid, *DHA* Docosahexanoic acid, *DPA* Docosapentaenoic acid, *DTA* Docosatetraenoic acid, *D5D* delta-5-Desatuase (C20:4 ω-6/C20:3 ω-6), *D6D* delta-6-Desatuase(C18:3 ω-6/C18:2 ω-6), *D9D* delta-9-Desatuase(C18:1 ω-9/C18:0), *EDA* Eicosadienoic acid, *EPA* Eicosapentaenoic acid, *GLA* γ-linolenic acid, *LA* Linoleic acid, *MUFA* monosaturated fatty acid, *PUFA* polysaturated fatty acid, *SFA* saturated fatty acid, C22:5 ω-6(Docosapentaenoic acid, DPA) = osbond acid; C22:5 ω-3 (Docosapentaenoic acid, DPA) = clupanodonic acid, *BMI* body mass index, *SBP* Systolic Blood Pressure, *DBP* Diastolic Blood Pressure, *TG* Triglyceride, *TC* total cholesterol, *HDL-C* high density lipoprotein cholesterol, *LDL-C* low density lipoprotein, cholesterol, *AI* (TC-HDL)/HDL, *Apo B* apolipoprotein B, *FBS* fasting blood sugar, *HOMA-IR* homeostasis model assessment of insulin resistance, *hs-CRP* high sensitivity C-reactive protein

### Comparison of cardiovascular risk factors in quantile subgroups of fatty acid indices

**Fig. 2:**
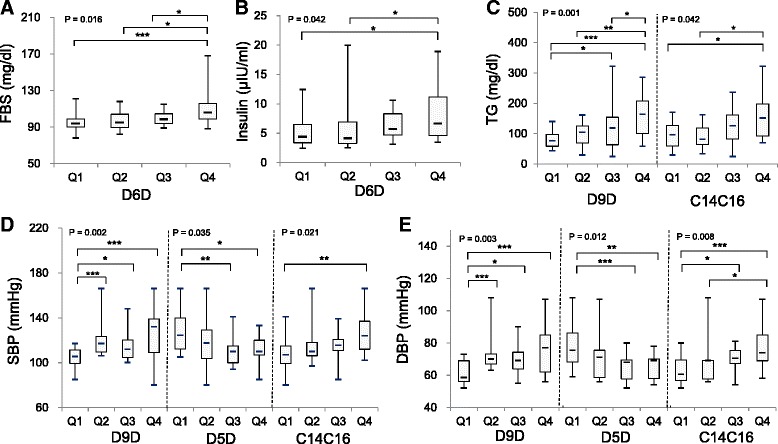
Comparison of cardiovascular risk factor levels in quartile subgroups of fatty acid indices. (**a**) fasting blood glucose (FBS) levels in the quartile groups of delta 6 desaturase index (D6D), (**b**) fasting insulin levels in the quartile groups of D6D index, (**c**) triglyceride (TG) levels in the quartile groups of delta 9 desaturase (D9D) index and the sum of C14:0 and C16:0 (C14C16), (**d**) systolic blood pressure (SBP) levels in the quartile groups of D9D, delta 5 desaturase index (D5D) and C14C16, and (**e**) diastolic blood pressure (DBP) levels in the quartile groups of D9D, D5D and C14C16

To examine the differences in levels of cardiovascular risk factors among the quantile subgroups of D9D, D5D, D6D, and the sum of C14:0 and C16:0 (C14:0 + C16:0) percentages, fatty acid indices were rank-transformed to generate subgroups as quantile groups. Significant differences among the subgroups of fatty acid indices were found in FBS, insulin, TG, SBP, and DBP (Fig. 2a–e). As concentrations of FBS and insulin increased, the D6D index values also increased. For instance, the levels of FBS or insulin in Q4 of D6D were significantly higher than those in Q1 (Fig. 2a, b). Serum TG levels were higher in the Q4 subgroups of D9D or C14:0 + C16:0 compared to Q1 of the corresponding indices (Fig. 2c). In both SBP and DBP quantile subgroups, the values of D9D and C14:0 + C16:0 in Q4 were higher than those of Q1 whereas the levels of D5D in Q4 were lower than that of Q1 (Fig. 2d, e). These results suggested that high D6D was associated with high FBS and/or insulin, high D9D and high C14:0 + C16:0 were related to high TG, and high D9D, high C14:0 + C16:0, and low D5D were associated with high blood pressures.

### Linear regression analysis using fatty acid indices and CVD risk factors

The relationship between fatty acid indices and FBS, insulin, TG, and blood pressures were examined after adjusting for sex, age, and BMI (Table 3). After adjustment, D6D remained significant in its association with FBS levels but not with insulin. Regardless of the adjustment, D9D and C14:0 + C16:0 were significantly associated with serum TG levels. However, SBP and DBP had altered associations with D9D, D5D, and C14:0 + C16:0 after the adjustment. Even if the individual index (D9D, D5D, or C14:0 + C16:0) was significant in its association with SBP or DBP, they failed to remain significant in a model containing all three indices. Interestingly, adjustment increased the explanatory powder of models for SBP or DBP and made D9D a significant contributor to SBP or DBP. These data suggested that D6D, D9D, and D9D and C14:0 + C16:0 demonstrated strong associations with FBS, blood pressures, and TG, respectively, after adjusting for sex, age, and BMI.

**Table 3:** Linear regression analysis with fatty acid indices and its associated risk factors

		R^2^	adjR^2^	B	S.E.	P
FBS	D6D	.150	.135	31.28	10.04	.003
	adj D6D	.278	.223	25.59	9.702	.011
Insulin	D6D	.082	.065	6.403	2.942	.034
	adj D6D	.172	.105	5.062	2.946	.092
TG	D9D	0.454	0.433	439.5	101.09	.000
	C14C16			23.76	5.561	.000
	adj D9D	0.518	0.471	496.7	102.4	.000
	adj C14C16			17.79	5.876	.004
SBP	D9D	0.211	0.166	54.88	34.27	.115
	D5D			−31.91	22.81	.168
	C14C16			3.720	2.025	.072
	adj D9D	0.355	0.277	77.61	33.46	.024
	adj D5D			−27.29	21.30	.206
	adj C14C16			1.560	2.025	.445
DBP	D9D	0.235	0.192	39.68	23.25	.094
	D5D			−22.56	15.47	.151
	C14C16			2.785	1.374	.048
	adj D9D	0.329	0.248	51.95	23.52	.032
	adj D5D			−20.01	14.97	.187
	adj C14C16			1.585	1.424	.271

Tested by linear regression; *adj* adjusted after age, *BMI* body mass index and sex; *FBS* fasting blood sugar, *TG* Triglyceride, *SBP* Systolic Blood Pressure, *DBP* Diastolic Blood Pressure, *D5D* delta-5-Desatuase (C20:4 ω-6/C20:3 ω-6), *D6D* delta-6-Desatuase (C18:3 ω-6/C18:2 ω-6), *D9D* delta-9-Desatuase (C18:1 ω-9/C18:0; *C14C16* the sum of C14:0 (myristic acid) + C16:0 (palmitic acid)

### Correlation between frequency of dietary intake of food groups and fatty acids in erythrocyte membrane phospholipids

There was a positive association between C16:1n7 and the vegetable and seaweed group (*r* = 0.437, *p* = 0.001). Dairy product intake showed a negative association with C20:3n6 and C22:4n6 (*r* = −0.333, *p* = 0.011 and *r* = −0.306, *p* = 0.020). The food group for tofu and eggs was positively associated with C20:5n3 (*r* = 0.314, *p* = 0.017). Other food groups, including meat, fish, shellfish, fruit, and rice, did not show any significant associations in the study subjects.

## Discussion

The study subjects were middle-aged men and women who were mostly over 50 years old, lived in the metropolitan area of South Korea, and were able to afford a special health check-up. The fatty acid patterns in the RBC membrane phospholipids in these subjects were investigated for their relationships with cardiovascular risk-associated parameters. After confirming the correlation between several individual fatty acids with cardiovascular risk-related factors, fatty acid indices were utilized to further investigate the relationships, including D6D, D5D, D9D, and C14:0 + C16:0. The current study was a retrospective clinical study and only a mini dietary assessment index for nutritional dietary intake analysis was available. The total energy, carbohydrate and protein intake, and alcohol consumption were estimated. Estimated alcohol consumption was adjusted for an analysis of the association of fatty acid indices with cardiovascular risk factors. In study subjects, the proportions of C20:5n3, C22:5n3, and C22:6n3 had no significant association with levels of cardiovascular risk factors. C20:5n3, C22:5n3, and C22:6n3 are long chain fatty acids (LCFAs) often used as markers of fish intake [14].

Based on the criteria for metabolic syndrome, 24.6 % of study subjects were centrally obese, and 22.4 % were obese according to BMI criteria (reference). BMI showed a positive association with C14:0. Waist circumference, another marker for abdominal obesity, was positively associated with C14:0 and C16:0 but negatively correlated with C18:2n6. These findings might indicate high dietary intake of SFAs and low intake of C18:2n6 in the subjects because C18:2n6 is not produced in the body. These results are similar to previous findings suggesting a positive association of obesity-related markers (BMI and waist circumference) with SFAs such as C16:0 and a negative association with C18:2n6 [11]. Therefore, lower dietary intake of C18:2n6 might be associated with increased BMI. In another study, sagittal abdominal diameter and waist girth were used as obesity-related markers. These markers showed strong positive association with D9D for C16:0 and D6D and a negative association with D5D [11]. This difference could be due to the relatively small number of subjects used in our study, which might have missed small changes in fatty acid patterns.

Alterations in serum lipid profiles are known risk factors for CVD. Of study subjects, 27 % had hypertriglyceridemia and 21 % had low HDL-C. AI and serum TG levels showed a positive relation with SFAs C14:0 and C16:0 whereas HDL-C was negatively associated with these markers. High saturated and/or animal fat diets increase serum TG levels [15]. Because palmitate (C16:0) is the most abundant SFA, the proportion of SFAs is likely to reflect that of C16:0. The sum of the proportions of C14:0 and C16:0 (C14:0 + C16:0) were used to further analyze its association with risk factors. The highest quartile groups of C14:0 + C16:0 showed significantly higher serum TG. C14:0 + C16:0 was a significant contributor to serum TG in linear regression analysis. Our findings support previous suggestions of a positive association between SFA and CVD.

We also noticed that serum TG had strong positive associations with C16:1n7 and C18:1n9. D9D catalyzes the production of C16:1n7 and C18:1n9 from C16:0 and C18:0, respectively [16]. The ratio of C18:1n9 to C18:0 was used to estimate D9D activity in this study. For serum TG and blood pressures, the highest quartile of D9D showed significant elevations in TG and blood pressures. In a linear regression analysis, D9D was a significant contributor to serum TG and blood pressures. The physiological role of D9D on regulation of adiposity, insulin resistance, and lipid synthesis has been implicated in an animal model [17]. Overexpression of D9D, which is usually referred to as stearoyl CoA desaturase-1 (SCD-1), showed an increase in TG production. A genetically modified mouse model lacking SCD-1 displayed significant reduction in serum TG [17, 18]. Based on these observations, the increase in D9D might reflect elevated production of TG in our study subjects. In our analysis of food groups, C16:1n7 was positively correlated with dietary intake of vegetables and seaweeds. This was unexpected considering the positive association between C16:1n7 and serum TG. These results warrant a further large scale study with detailed dietary intake to verify.

Other cardiovascular risk factors, including ApoB, blood pressures, HOMA-IR, and homocysteine, were also positively correlated with C18:1n9. Inconsistent with our findings, an increased incidence of left ventricular hypertrophy and cardiovascular risk factors were shown to be strongly associated with increased SFA and C18:1n9 in serum cholesterol esters [19].

Interestingly, high blood pressures are significantly associated with high D9D and low C24:1n9. Changes in D9D were closely associated with blood TG, which might indirectly influence blood pressures. C24:1n9 levels in erythrocyte membrane fatty acids were highly positively associated with dietary PUFA intake along with C18:3n3 and C18:2n6 [20]. Inverse relations between C24:1n9 and blood pressures suggests that low dietary PUFA intake in our subjects was related to high blood pressures.

C18:1n9 was positively associated with both serum TG and blood pressures, but C20:1n9 and C24:1n9 had negative associations with these risk factors. These findings suggest that monounsaturation in long chain fatty acids with fewer than 20 carbon atoms (<20 carbon-LCFAs) was likely associated with high TG and high blood pressure while monounsaturation in >20 carbon-LCFAs was inversely related to serum TG and blood pressures.

C20:4n6 and C20:5n3 are produced by series action of D6D, elongase and D5D [21, 22]. A high ratio of C20:4n6 to C20:5n3 (AA/EPA ratio) is related to the risk of cardiovascular disorders [23]. C20:4n6 is a substrate for the enzymes for the production of pro-inflammatory cytokines and C20:5n3 competes with C20:4n6 [23]. On the other hand, a low ratio of C20:4n6 to C20:3n6 was previously detected prior to the development of myocardial infarctions in middle-aged men [8], suggesting that low D5D (C20:4n6/C20:3n6) might be related to CVD risk factors. In our study, D5D showed a negative association with serum TG, blood pressure, ApoB, and AI. Low C20:4n6 was related to high levels of serum TG, blood pressures, and ApoB. However, only blood pressure levels were significantly different for D5D quartile groups. In univariate linear regression, D5D was a significant contributor to blood pressure but did not remain significant with multivariate linear regression analysis. This suggests that the association of D5D with blood pressures did not attain statistical significance after the adjustment of BMI, age and sex. Instead, D9D appeared to be statistically associated with blood pressure in that analysis. The high content of C22:6n3 (docosahexaenoic acid, DHA) was shown to exert cardiovascular protective effects [24]. However in our study we didn’t see a statistical association with clinical parameters related to cardiovascular disorders. Our data may provide evidence to support that high D9D is associated with elevated blood pressures.

Diabetes-associated risk factors, including FBS, insulin, and HOMA-IR were strongly associated with C14:0 and C18:3n6, a product generated by D6D. D6D also showed positive correlations with FBS, HbA1c, and HOMA-IR. The highest quartile of D6D had the highest levels of FBS. In linear regression analysis, adjusted D6D remained significant for FBS, indicating that D6D might contribute to FBS. Our findings are in line with previous reports showing that insulin resistance was associated with an increase in D6D and C18:3n6 [25, 26]. In a 10 year follow-up study, subjects who developed non-insulin-dependent diabetes mellitus (NIDDM) had high SFA and C18:3n6 [26]. Apart from D6D, low C18:2n6 has been implicated in insulin resistance and metabolic syndrome [27]. Other studies have reported that impaired fasting glycemia and diabetes were associated with high SFA and low C18:2n6 [28]. High D9D has been implicated in insulin resistance and diabetic conditions [27, 29]. Insulin sensitivity was improved in an animal model with deficient D9D [17]. These findings indicate that low D6D, low D9D, and high C18:2n6 are associated with decreased risk of diabetes and associated conditions.

## Conclusions

Here, we provided evidence that the fatty acid patterns of erythrocyte membrane phospholipids are strongly associated with CVD risk factors in middle aged Koreans. In particular, high serum TG is associated with high D9D and C14:0 + C16:0. FBS was positively associated with D6D. SBP and DBP showed strong positive associations with D9D. Because our study was a retrospective cross-sectional study, we could not infer the cause and effect relationship between fatty acid indices and CVD risk factors. Specific associations found in our study warrant further large scale studies involving a greater number of subjects.

## Methods and materials

### Subjects

All the data from patients were retrieved from a registry upon approval by Chung-Ang University Institutional Review Board (IRB) (IRB number C2014199 (1396)). Patients who underwent a specialized health examination from January 2014 until January 2015 at Chung-Ang University Hospital Examination Centre were initially assessed for eligibility. Inclusion criteria were age between 40 years and 65 years, anthropometry measurements, blood biochemistry, erythrocyte membrane fatty acid composition, and dietary intake assessment. From 62 participants in total, five patients were excluded due to missing measurement data. Chung-Ang University IRB approved all procedures for this research.

### Anthropometric measurements

A body composition analyzer (Inbody720®, Biospace, Korea) was used to measure height (cm), weight (kg), body fat percentage (%), and waist circumference (cm). Body mass index (BMI) was measured using the obtained height and weight. Blood pressure was measured from the arms using an automatic blood pressure monitor (HEM – 1000, OMRON, Japan) after patients had rested for 10 minutes and a steady heart rate was achieved. The values were averaged from two readings of systolic and diastolic blood pressures.

### Biochemical analysis

Blood tests were carried out after 10 hours of fasting. Three ml of blood obtained from the forearm vein (cephalic vein, basilica vein) was collected in a vacuum blood collection bottle. Blood was treated with 3 % EDTA and centrifuged at 4 °C, 3000 rpm for 15 minutes to separate the plasma. Separated plasma was stored at −20 °C until analysis. Blood levels of fasting blood glucose (FBS), HbA1c, total cholesterol (Total-C), HDL cholesterol (HDL-C), LDL cholesterol (LDL-C), triglycerides (TG), aspartate aminotransferase (AST), alanine aminotransferase (ALT), homocysteine, and apolipoprotein B (ApoB) were assessed using an automatic chemistry analyzer (ADI-VA 1650 Chemistry System, Bayer, Tarrytown, NY, USA). Hypersensitive C reactive peptide (hs-CRP) was measured using COBAS INTEGRA 700 (Roche Diagnostic System, Basel, Switzerland) with the particle enhanced immunoturbidometric method. Artherogenic index (AI) was calculated using the formula ‘(Total-C-HDL-C)/HDL-C’ [30]. Insulin resistance was calculated using the formula ‘fasting insulin (U/ml) x fasting plasma glucose (mmol/L) / 22.5’ based on the homeostasis model assessment (HOMA) method [31].

### Red blood cell membrane fatty acid analysis

After centrifugation of blood in an EDTA-treated tube at 4 °C, 3000 rpm for 15 minutes, red blood cells were separated from plasma. Red blood cells were washed three times using phosphate buffered saline (PBS pH7.2). The total volume of red blood cells was doubled by adding PBS to the RBC suspension, and then samples were stored frozen at −20 °C until analysis. As an internal standard to measure lipid weight, 100 μg of heptadecanoic acid (17:0) was added to 200 ml of plasma. The extraction of total lipids from RBC and plasma was carried out using ice-cold chloroform/methanol (2:1, v/v) containing 0.01 % (w/v) butylated hydroxytoluene (BHT) by the Folch method [32]. The Lepage and Rog method was used to generate methyl ester forms of fatty acids [33]. Prior to lipid extraction from RBCs, the RBCs were saponified and non-saponified fats were removed using the modified method generated by Tilvis and Miettinen [34]. Lipid extraction from saponified RBCs was carried out by the same method described for plasma. Fatty acid methyl esters were separated and analyzed by Shimadzu GC-9A gas chromatography (Shimadzu Corp., Kyoto, Japan) equipped with a flame ionization detector and a fused silica capillary column (FS-WCOT, 30 m × 0.2248 mm id 0.15 μm film thickness). The injector and detector temperature was 270 °C. Helium gas was used as a carrier gas at a flow rate of 25 ml/min and split ratio of 1:100. Fatty acid methyl esters were identified by comparing their retention times with those of standards purchased from Sigma. The activities of delta 5 desaturase (D5D), delta 6 desturase (D6D), and delta 9 desaturase (D9D) were estimated using the ratios of C20:4n-6 and C20:3n-6 for D5D activity, C18:3n-6 and C18:2n-6 for D6D activity, and C18:1 and C18:0 for D9D activity [25, 35]. Total SFA was the sum of the percentage of C14:0, C16:0, C18:0, and C24:0. Total MUFA was the sum of C16:1n7, C18:1n9, C20:1n9, and C24:1n9. Total PUFA was the sum of C18:2n6, C18:3n6, C20:2n6, C20:3n6, C20:4n6, C22:4n6, C22:5n6, C18:3n6, C20:5n3, and C22:5n3.

### Dietary intake questionnaire

To assess diet quality, a modified version of the mini dietary assessment index for Korean adults (K-MDA) developed by Kim et al. [36] was used. The questionnaire consisted of 10 questions from K-MDA and an additional 10 questions. Each question is shown in Table 1. The 20 questions were assessed using a 5-point Likert scale (1: Never, 3: Sometimes, 5: Always) with a total of 100 points. Five points refers to desirable eating habits, while 3 points and 1 point refer to normal eating habits and unhealthy eating habits that require improvement, respectively. For questions 1–7, 13,16, and 18–20, answers were worth ‘Always’ - 5 points, ‘Sometimes’ – 3 points, and ‘Never’ – 1 point. For questions 8, 14, 15, 16, and 17, each answer was worth ‘Always’ - 1 point, ‘Sometimes’ – 3 points, and ‘Never’ – 5 points. The total score was then calculated. Higher total scores indicate healthier eating habits.

### Statistical analysis

All data were statistically analyzed using SPSS Version 21.0 (SPSS Inc., Chicago, IL, USA). The values were expressed as mean and standard deviation (SD). To compare men and women, the study variables were examined by independent t-test if variables demonstrated normal distribution or by Mann–Whitney U test if variables were not normally distributed. Normal distribution was assessed by the Shapiro-Wilk test. Relationships among BMI, blood pressures, blood chemistry, and proportions of fatty acids were tested using Spearman’s rank correlations. To evaluate the relationships between fatty acid indices and cardiovascular risk factors, D9D, D5D, D6D, and C14:0 + C16:0 values were rank-transformed and classified into categorical variables: Q1 (below 25 % quantile), Q2 (between 25 and 50 %), Q3 (between 50 and 75 %), and Q4 (above 75 % quantile). Differences in the values of cardiovascular risk factors among categorical groups were evaluated using a nonparametric Kruskal-Wallis test followed by a nonparametric two-tailed Mann–Whitney test. Univariate relationships between cardiovascular risk factors and fatty acid indices, including D9D, D5D, D6D, M/S ratio, and C14:0 + C16:0 were tested using simple linear regression analysis adjusted for sex, age, and BMI. In these analyses, D9D, D5D, and D6D were logarithmically transformed. A value of *p* <0.05 was considered statistically significant.

## Additional file

Additional file 1: Table S1. Fatty acid compositions in the male and female subjects in the study. (DOCX 16 kb)

## Abbreviations

D9D: Delta 9 desaturase
D6D: Delta 6 desaturase
D5D: Delta 5 desaturase
CVD: Cardiovascular disorders
TG: Triglyceride
SBP: Systolic blood pressure
DBP: Diastolic blood pressure groups
FBS: Fasting blood glucose
hsCRP: High sensitive C-reactive peptide
AI: Atherogenic index
ApoB: Apolipoprotein B
SFA: Saturated fatty acids
PUFA: Polyunsaturated fatty acids
MUFA: Monounsaturated fatty acids
C14:0 + C16:0: the sum of the percentage of C14:0 and C16:0
BMI: Body mass index
SCD-1: Stearoyl CoA desaturase-1
