# Supplementary material for: The association of cardiovascular risk factors with saturated fatty acids and fatty acid desaturase indices in erythrocyte in middle-aged Korean adults
Source: Lipids Health Dis. 2015 Oct 24;14:133. doi: 10.1186/s12944-015-0135-x (PMC4619393; doi:10.1186/s12944-015-0135-x)
Supplement: Additional file 1: Table S1. — Fatty acid compositions in the male and female subjects in the study. (DOCX 16 kb) [file 12944_2015_135_MOESM1_ESM.docx]

Additional file 1: Table S1. Fatty acid compositions in the male and female subjects in the study

|  | Male (n=35) | Female (n=22) | *p* value |
| --- | --- | --- | --- |
|  |  |  |  |
| C14:0 Myristic acid | 0.534 ± 0.153 | 0.471 ± 0.100 | 0.065^§^ |
| C16:0 Palmitic acid | 23.25 ± 1.142 | 22.83 ± 0.872 | 0.151 |
| C18:0 Stearic acid | 14.18 ± 0.754 | 14.15 ± 1.063 | 0.901 |
| C24:0 Lignoceric acid | 0.494 ± 0.229 | 0.380 ± 0.108 | 0.007^§^ |
| C16:1n7, Palmitoleic acid | 0.937 ± 0.295 | 1.009 ± 0.391 | 0.640^§^ |
| C18:1n9, Oleic acid | 16.77 ± 1.310 | 16.52 ± 2.149 | 0.248^§^ |
| C20:1n9, Eicosenoic acid | 0.202 ± 0.748 | 0.196 ± 0.629 | 0.987^§^ |
| C24:1n9, Nervonic acid | 0.415 ± 0.153 | 0.397 ± 0.193 | 0.385^§^ |
| C18:2n6 (LA) | 15.68 ± 1.674 | 16.77 ± 1.403 | 0.014 |
| C18:3n6 (GLA) | 0.156 ± 0.050 | 0.154 ± 0.070 | 0.549^§^ |
| C20:2n6 (EDA) | 0.342 ± 0.093 | 0.341 ± 0.102 | 0.593^§^ |
| C20:3n6 (DGLA) | 1.410 ± 0.222 | 1.341 ± 0.311 | 0.337 |
| C20:4n6 (AA) | 11.11 ± 1.378 | 10.99 ± 1.322 | 0.736 |
| C22:4n6 (DTA) | 1.275 ± 0.279 | 1.655 ± 2.453 | 0.294^§^ |
| C22:5n6 (DPA) | 0.389 ± 0.124 | 0.340 ± 0.106 | 0.133 |
| C18:3n3 (ALNA) | 0.403 ± 0.130 | 0.456 ± 0.121 | 0.126 |
| C20:5n3 (EPA) | 1.883 ± 0.551 | 1.835 ± 0.726 | 0.451^§^ |
| C22:5n3 (DPA) | 1.939 ± 0.238 | 1.838 ± 0.208 | 0.127^§^ |
| C22:6n3 (DHA) | 7.351 ± 0.723 | 7.449 ± 1.143 | 0.695 |
| D5D | 8.086 ± 1.641 | 8.686 ± 2.451 | 0.302^§^ |
| D6D | 0.010 ± 0.003 | 0.009 ± 0.004 | 0.420^§^ |
| D9D | 1.188 ± 0.139 | 1.181 ± 0.230 | 0.491^§^ |

Mean±S.D.; Tested by independent t test.(^§^ is mann-whitney U test). AA: Arachidonic acid, ALNA:

α-Linolenic acid, DGLA: Dihomo-γ-linolenic acid, DHA: Docosahexanoic acid, DPA: Docosapentaenoic

acid, DTA: Docosatetraenoic acid, D5D: delta-5-Desatuase (C20:4 ω-6/C20:3 ω-6),

D6D: delta-6-Desatuase(C18:3 ω-6/C18:2 ω-6), D9D: delta-9- Desatuase(C18:1 ω-9/C18:0),

EDA: Eicosadienoic acid, EPA: Eicosapentaenoic acid, GLA: γ-linolenic acid, LA: Linoleic acid,

MUFA: monosaturated fatty acid, PUFA: polysaturated fatty acid, SFA: saturated fatty acid,

C22:5 ω-6(Docosapentaenoic acid, DPA) = osbond acid, C22:5 ω-3 (Docosapentaenoic acid,

DPA) = clupanodonic acid.
